# LncRNA TUG1 Repressed Angiogenesis by Promoting the Ubiquitination of HuR and Inhibiting Its Nuclear Translocation in Cerebral Ischemic Reperfusion Injury

**DOI:** 10.1002/advs.202413333

**Published:** 2025-01-31

**Authors:** Hongxiang Jiang, Qiang Cai, Peidong He, Fei Li, Qianxue Chen

**Affiliations:** ^1^ Department of Neurosurgery Renmin Hospital of Wuhan University Wuhan Hubei Province 430060 China; ^2^ First School of Clinical Medicine of Wuhan University Wuhan Hubei Province 430060 China

**Keywords:** angiogenesis, cerebral Ischemic reperfusion Injury, HuR, TUG1, VEGFA mRNA

## Abstract

Although both Taurine Upregulated Gene 1(TUG1) and Human Antigen R (HuR) play significant regulatory roles in Cerebral Ischemic Reperfusion Injury (CIRI), their potential pro‐angiogenesis mechanisms in CIRI remain unclear. Methods: Herein, the biological roles of TUG1 and HuR in angiogenesis are first confirmed. Following that, HuR‐binding VEGFA mRNAs are identified via the Fluorescence In Situ Hybridization (FISH), RNA Immunoprecipitation (RIP), and Cross‐Linking Immunoprecipitation (CLIP) assays. Actinomycin D and polysomal assays are also employed to confirm VEGFA mRNA stability. The co‐localization of TUG1 with HuR is confirmed using FISH, while the RIP and RNA pull‐down assays are employed to elucidate their interplay. The direct binding between TUG1 and HuR is confirmed through the CLIP assay. Co‐Immunoprecipitation (Co‐IP) and rescue experiments are performed to further elucidate TUG1‐HuR interactions. Results: While TUG1 repressed angiogenesis and aggravated CIRI, HuR exerted contrary effects. Specifically, HuR bound directly to VEGFA mRNA, a phenomenon that enhanced VEGFA mRNA stability. Conversely, TUG1 binds to HuR directly, inhibiting its nuclear translocation and promoting its ubiquitination, ultimately reducing VEGFA mRNA stability. Conclusions: It is found that TUG1 can inhibit angiogenesis in CIRI through the HuR/VEGFA mRNA axis.

## Graphical abstract

TUG1 inhibited angiogenesis through the HuR/VEGFA mRNA axis in CIRI. Upon ischemic stroke, TUG1 expression is up‐regulated in HUVECs. TUG1 targeted to HuR by promoting its ubiquitination and inhibiting its nuclear translocation, subsequently restricting the stability of VEGFA mRNA and inhibiting HUVECs angiogenesis.

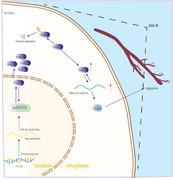

## 1 Introduction

Ischemic Stroke (IS) accounts for a significant proportion of mortalities and disability incidences globally, highlighting its profound and extensive impact on public health.^[^ ^1^, ^2^ ^]^ Presently, IS interventions primarily involve blood circulation recovery, a process that could lead to Cerebral Ischemic Reperfusion Injury (CIRI), whose pathogenesis encompasses the exacerbation of hypoxia‐ and ischemia‐induced nerve damage following blood flow restoration.^[^ ^3^ ^]^ Notably, increased micro‐vessel density of collateral circulation could improve clinical prognosis post‐CIRI.^[^ ^4^ ^]^ Therefore, promoting post‐ischemic angiogenesis to rapidly rebuild blood circulation in an ischemic area could be a promising strategy for restorative CIRI treatment.^[^ ^5^ ^]^

Long non‐coding RNAs (lncRNAs), a prominent class of RNAs with a size > 200 nucleotides and with no protein‐coding ability, participate in various biological processes by controlling gene/protein expression, stability, and activation.^[^ ^6^, ^7^, ^8^ ^]^ For instance, Taurine Upregulated Gene 1 (TUG1) has been associated with pathologic angiogenesis. Specifically, TUG1 was found to promote tumor‐induced angiogenesis in glioblastoma.^[^ ^9^ ^]^ Furthermore, TUG1 could suppress miR2045p and activate the downstream signaling pathway of JAK2/STAT3, thus facilitating angiogenesis in hepatoblastoma.^[^ ^10^ ^]^ Although the regulatory role of TUG1 in CIRI within neuronal cells is well established in the literature,^[^ ^11^ ^]^ the specific roles and mechanisms of TUG1 in angiogenesis post‐CIRI remain unclear.

According to research, RNA‐Binding Proteins (RBPs), master regulators of RNA fate from synthesis to decay, are responsible for RNA processing (splicing, capping, and polyadenylation), transport, degradation, and translation.^[^ ^12^ ^]^ For instance, Human Antigen R (HuR), a ubiquitous member of the RBP family, binds to transcripts in AU‐rich elements, contributing to the stability of target mRNAs.^[^ ^13^, ^14^ ^]^ Additionally, previous animal studies reported HuR downregulation in cerebral hemispheres of mice experiencing temporary blockage of the Middle Cerebral Artery (MCA).^[^ ^15^ ^]^ Furthermore, HuR was reported to be crucially involved in angiogenic edema in mice CIRI models, implying that it could regulate angiogenesis.^[^ ^16^ ^]^ These research insights suggest that HuR can significantly regulate Vascular Endothelial Cell (VEC) proliferation, highlighting its potential involvement in various CIRI‐related pathological processes. However, the role of HuR in CIRI is yet to be fully elucidated.

Besides regulating precursor splicing, as well as mRNA stabilization, localization, and translation, HuR could also bind to various lncRNAs, exerting a series of biological effects.^[^ ^17^ ^]^ Therefore, like Transcription Factors (TFs), LncRNAs may be employed to bind, store, or sequester molecules to particular subcellular locations. Moreover, some lncRNAs may function as “sponges” for RBPs.^[^ ^18^ ^]^ Overall, although both TUG1 and HuR play significant regulatory roles in CIRI, their potential pro‐angiogenesis mechanisms in CIRI remain unclear, forming the basis of this study.

## 2 Results

### 2.1 TUG1 Inhibited Angiogenesis and Aggravated CIRI both In Vivo and In Vitro

**Figure 1:**
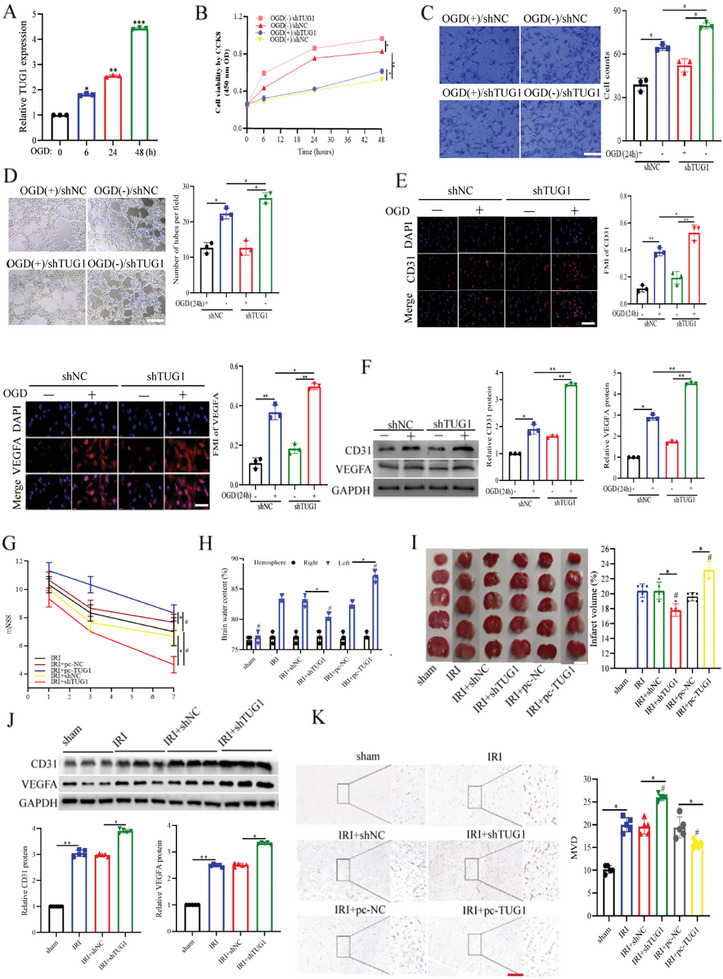
TUG1 inhibited angiogenesis and aggrevated CIRI in *vivo* and in vitro. A) The level of TUG1 was analyzed after OGD treatment by RT‐qPCR (*n* = 3). B–D) The proliferation, migration, and tube formation ability were measured respectively (*n* = 3). Scar bar = 25um. E) The levels of CD31 and VEGFA in HUVECs were detected by the immune‐fluorescence (*n* = 3). Scar bar = 25um. F) The CD31 and VEGFA levels were measured by WB (*n* = 3). G) Time‐course of modified neurological severity scores. # *p* < 0.05 versus IRI group (*n* = 5). H) The measured brain water content. # *p* < 0.05 versus IRI group (*n* = 5). I) The TTC‐stained mice brain slices. # *p* < 0.05 versus IRI group (*n* = 5). Scale bars = 5 mm. J) The brain tissue's CD31 and VEGFA levels were detected by WB (*n* = 5). K) The microvessel density was detected by the immunohistochemical analysis (*n* = 5). Scar bar = 25um. Data are presented as means ± SD. ^*^p < 0.05, ^**^p < 0.01 as calculated by Mann–Whitney test (for G and I) and one‐way ANOVA with Bonferroni's multiple comparison post hoc test (for A–F,H–K).

Herein, we found that TUG1 significantly increased HUVEC proliferation in a time‐dependent manner following OGD treatment and 24 h of reoxygenation (**Figure** **1**A). After successfully constructing HUVEC models with TUG1 knockdown or overexpression (Figure S1A, Supporting Information), we observed that TUG1 knockdown enhanced HUVEC proliferation, migration, and tube formation abilities (Figure 1B–D). Moreover, the ELISA results revealed that compared to the control group, the OGD/R group exhibited significantly higher levels of VEGFA in the supernatant of HUVECs (Figure S1B, Supporting Information). Furthermore, the IF assays revealed CD31 and VEGFA upregulation following TUG1 knockdown (Figure 1E). In other words, TUG1 knockdown significantly upregulated the CD31 and VEGFA proteins (Figure 1F). Notably, TUG1 overexpression yielded contrary results (Figure S1C–G, Supporting Information). These findings collectively suggest that TUG1 knockdown suppressed angiogenesis in OGD/R‐treated HUVECs.

Compared to the sham group, CIRI mice exhibited higher TUG1 expression in the cerebral cortex (Figure S2A, Supporting Information). As outlined in the time schedule, mice with TUG1 knockdown, TUG1 overexpression, or both were first confirmed in vivo (Figure S2B,C, Supporting Information). Subsequently, CIRI‐induced mice were subjected to mNSS, revealing that TUG1 overexpression aggravated the neurological deficit (Figure 1G). Furthermore, mice with TUG1 overexpression exhibited more cerebral edema and bigger infarct volumes (Figure 1H,I). Moreover, the CD31 and VEGFA protein levels decreased significantly following TUG1 overexpression (Figure 1J). The Immunohistochemical (IHC) analysis results also revealed that vessel density dropped after TUG1 overexpression (Figure S2D, Supporting Information). These findings collectively suggest that TUG1 inhibited angiogenesis and aggravated CIRI.

### 2.2 HuR Promoted Angiogenesis and Attenuated CIRI both In Vivo and In Vitro

**Figure 2:**
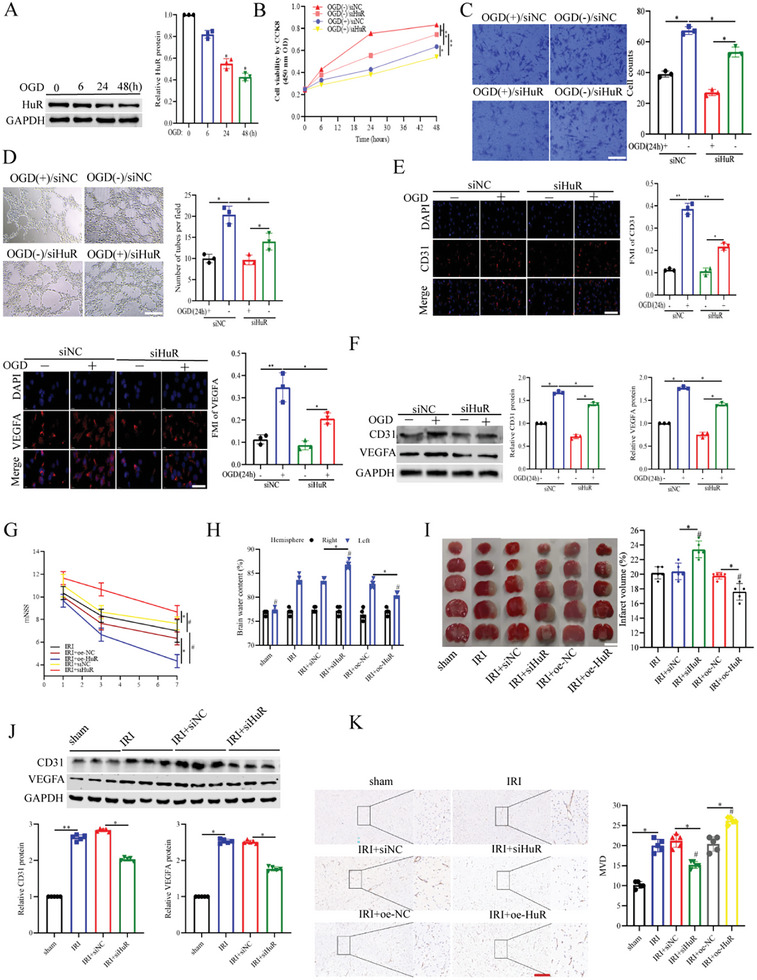
HuR promoted angiogenesis and attenuated CIRI in *vivo* and in vitro. A) The level of HuR was analyzed after OGD treatment by WB (*n* = 3). B–D) The proliferation, migration, and tube formation ability were measured respectively (*n* = 3). Scar bar = 25um. E) The levels of CD31 and VEGFA in HUVECs were detected by the immune‐fluorescence (*n* = 3). Scar bar = 25 um. F) The CD31 and VEGFA levels were measured by WB (*n* = 3). G) Time‐course of modified neurological severity scores. # *p* < 0.05 versus IRI group (*n* = 5). H) The measured brain water content. # *p* < 0.05 versus IRI group (*n* = 5). I) The TTC‐stained mice brain slices. # *p* < 0.05 versus IRI group (*n* = 5). Scale bars = 5 mm. J) The brain tissue's CD31 and VEGFA levels were detected by WB (*n* = 5). K) The microvessel density was detected by the immunohistochemical analysis (*n* = 5). Scar bar = 25 um. Data are presented as means ± SD. ^*^p < 0.05, ^**^p < 0.01 as calculated by Mann–Whitney test (for G and I) and one‐way ANOVA with Bonferroni's multiple comparison post hoc test (for A–F, H–K).

After culturing across different periods with OGD and reoxygenation for 24 h, we found that HuR expression in HUVECs decreased in a time‐dependent manner (**Figure** **2**A). Furthermore, after successfully constructing cell models with HuR knockdown or overexpression (Figure S3A,B, Supporting Information), we assessed the effects of HuR on the cells. In the OGD/R group, HuR knockdown reduced HUVEC proliferation, migration, and tube formation abilities (Figure 2B–D). Additionally, the IF assay results revealed that HuR knockdown decreased CD31 and VEGFA expression (Figure 2E). Moreover, treatment with HuR knockdown plasmids reversed the OGD/R treatment‐induced CD31 and VEGFA upregulation in HUVECs (Figure 2F). Notably, HuR overexpression yielded contrary results (Figure S3C–G, Supporting Information). These findings collectively suggest that HuR promoted angiogenesis in OGD/R‐treated HUVECs.

To explore the changes in HuR patterns in CIRI mice as described in the above‐mentioned time schedule, we successfully constructed an MCAO/R model (Figure S4A,B, Supporting Information). Compared to the sham group, the MCAO/R group showed lower HuR mRNA and protein levels (Figure S4C,D, Supporting Information). Subsequently, mice with HuR knockdown, HuR overexpression, or both were confirmed in vivo (Figure S4E,F, Supporting Information). HuR knockdown aggravated the neurological deficit, HuR overexpression exerted a contrary effect (Figure 2G). Furthermore, mice with HuR knockdown exhibited more cerebral edema and bigger infarct volumes, while mice with HuR overexpression showed an opposite phenomenon (Figure 2H,I). Moreover, HuR knockdown resulted in a significant drop in CD31 and VEGFA protein expression, which increased after HuR overexpression (Figure 2J). Additionally, according to the IHC analysis results, HuR knockdown decreased vessel density, (Figure 2K), with HuR overexpression yielding contrary results (Figure S4G, Supporting Information). These findings collectively suggest that HuR promoted angiogenesis and attenuated CIRI.

### 2.3 HuR Promoted Angiogenesis by Targeting VEGFA mRNA

**Figure 3:**
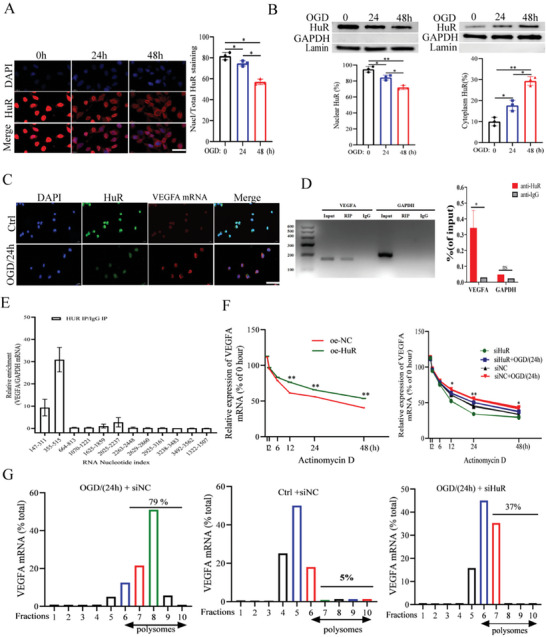
HuR promoted angiogenesis by targeting VEGFA mRNA. A) Immunoblotting detection of HuR protein in HUVECs after exposing to OGD for various time points (*n* = 3). Scale bar = 25 um. B) HuR protein expression in cytoplasmic and nuclear fraction of HUVECs (*n* = 3). GAPDH was used as control for cytoplasmic fraction, and Lamin was used here for nuclear fraction. C) Cellular distribution of HuR (green) and VEGFA mRNA (red) were detected by FISH (*n* = 3). Nuclei were stained with DAPI (blue). Scale bar = 50 um. D) The target binding between HuR and VEGFA mRNA was detected with RIP assay (*n* = 3). E) The binding sites between HuR and VEGFA mRNA was detected with CLIP assay (*n* = 3). F) VEGFA mRNA stability was measured by RT‐qPCR after HUVECs were exposed to Actinomycin D (5 mg mL^−1^), with control or OGD/24h, scramble or siRNA against HuR, and HUVECs with HuR overexpression construct or control vector. The data are expressed as the percentage of mRNA molecules before the actinomycin D treatment (*n* = 3). G) Polysomes assays were performed in HUVECs. VEGFA mRNA was measured with Taqman probes and presented here as the percentage in each fraction (*n* = 3). ^*^ *p* < 0.05, ^**^ *p* < 0.01 as calculated by one‐way ANOVA with Bonferroni's multiple comparison post hoc test.

To explore the potential angiogenesis mechanisms of HuR, the HUVECs were subjected to short‐term OGD culturing followed by reoxygenation for 24 h. According to the results, HuR shifted to the cytosol from the nucleus in a time‐dependent manner (**Figure** **3**A). Furthermore, cellular fractionation experiments revealed that the nuclear‐to‐cytoplasmic ratio of HuR decreased following OGD/R treatment (Figure 3B). These findings suggest that OGD/R treatment could induce HuR to translocate from the nucleus to the cytosol.

According to the FISH analysis results, HuR colocalized with VEGFA mRNA (Figure 3C). Subsequently, target binding between HuR and VEGFA mRNA was confirmed using the RIP assay (Figure 3D). The CLIP assay was also performed, demonstrating that the binding of HuR with VEGFA mRNA was mainly enriched at sites 355–515 (Figure 3E). Additionally, VEGFA mRNA levels were detected in HUVECs following actinomycin D treatment, revealing that HuR knockdown decreased the half‐life of VEGFA mRNA, while HuR overexpression exerted a contrary effect (Figure 3F). Lastly, the polyribosome assays revealed that only 5% of VEGFA mRNA correlated with polysomal fractions in untreated HUVECs, with OGD/R‐treated HUVECs showing an enhanced proportion (79%). Meanwhile, upon HuR silencing, the abundance in the polysomal fraction dropped to 37% (Figure 3G). These findings collectively suggest that HuR promoted angiogenesis by binding to VEGFA mRNA and enhancing its stability.

### 2.4 TUG1 Interacted with HuR

**Figure 4:**
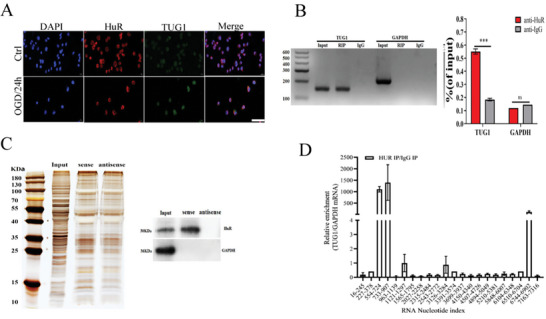
TUG1 interacted with HuR. A) Cellular distribution of TUG1(green) and HuR (red) were detected by FISH (*n* = 3). Nuclei were stained with DAPI (blue). Scale bar = 50 um. B) In up‐regulated HUVECs, an Anti‐HuR antibody was used for immune and co‐precipitation. TUG1 was detected in both Input group and the RIP group, and there was no significant difference with the negative control (*n* = 3). C) The direct binding between HuR and TUG1 was detected with RNA pull‐down and WB (*n* = 3). D) The binding sites between TUG1 and HuR was detected with CLIP (*n* = 3). ^*^ *p* < 0.05, ^**^ *p* < 0.01 as calculated by Mann–Whitney test (for B) and one‐way ANOVA with Bonferroni's multiple comparison post hoc test (for E).

To further establish whether TUG1 interacted with HuR in OGD/R‐induced HUVECs, we first conducted subcellular localization analysis using the FISH assay, revealing TUG1‐HuR colocalization in HUVECs (**Figure** **4**A). Furthermore, the RIP assays revealed that TUG1 could bind to HuR directly, a phenomenon the RNA pull‐down assay also confirmed (Figure 4B,C). Moreover, the CLIP assays revealed that the direct binding between TUG1 and HuR was mainly enriched at sites 554–724, 733–907, and 6744–6902 (Figure 4D). These findings collectively confirm TUG1‐HuR binding.

### 2.5 TUG1 Inhibited the Nuclear Translocation of HuR

**Figure 5:**
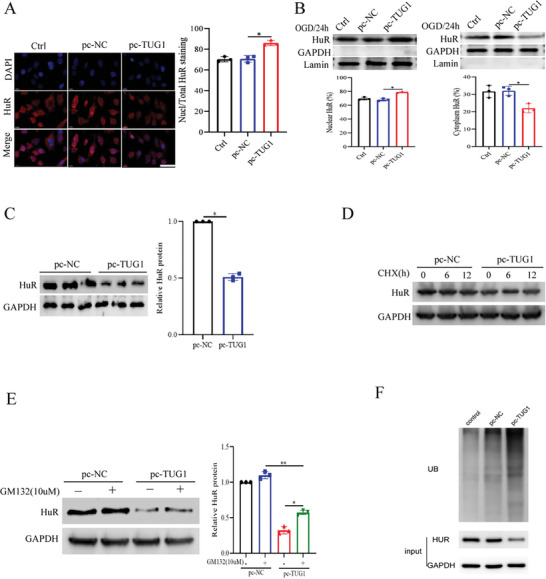
TUG1 inhibited the nuclear translocation and promoted the ubiquitination degradation of HuR. A) Immunoblotting detection of HuR protein in HUVECs with the up‐regulation of TUG1 (*n* = 3). Scale bar = 25 um. B) HuR protein expression in cytoplasmic and nuclear fraction of HUVECs (*n* = 3). GAPDH was used as control for cytoplasmic fraction, and Lamin was used for nuclear fraction. C) The expression of HuR in HUVECs transfected with pc‐TUG1 was detected by WB (*n* = 3). D) Stability of HuR protein in HUVECs with up‐regulation of TUG1 evaluated by cycloheximide chase assays (*n* = 3). E) Protein expression of HuR in HUVECs treated with proteasome inhibitor MG132 after transfection with pc‐TUG1 (*n* = 3). F) Immunoprecipitation analysis for ubiquitination modification of HuR in HUVECs with the up‐regulation of TUG1 (*n* = 3). ^*^ *p *< 0.05, ^**^ *p* < 0.01 as calculated by one‐way ANOVA with Bonferroni's multiple comparison post hoc test.

To further explore the potential angiogenesis mechanisms of TUG1, the FISH assay, and subcellular fractionation analysis were conducted, revealing that both the nucleus and cytoplasm exhibited TUG1 expression, most predominantly in the nucleus. Notably, OGD/R treatment could not significantly alter TUG1 expression in the nucleus (Figure S5A,B, Supporting Information). Furthermore, TUG1 overexpression inhibited the nucleocytoplasmic shutting of HuR in HUVECs (**Figure** **5**A). Moreover, the cellular fractionation experiments demonstrated that compared to the control groups, the nucleus‐cytoplasmic ratio of HuR was higher following OGD/R treatment (Figure 5B). These findings suggested that TUG1 inhibited the nuclear translocation of HuR in OGD/R‐induced HUVECs.

### 2.6 TUG1 Promoted the Ubiquitination Degradation of HuR

Interestingly, unlike at the mRNA level, TUG1 overexpression significantly downregulated HuR at the protein level (Figure 5C; Figure S5C, Supporting Information), suggesting that TUG1 might post‐transcriptionally regulate HuR expression. To assess the stability of the HuR protein, we treated HUVECs with TUG1 overexpression with CHX, revealing a significantly reduced stability of the HuR protein following TUG1 overexpression (Figure 5D). This finding implies that TUG1 might be involved in HuR protein degradation in HUVECs.

The two primary mechanisms for protein degradation in cells are the autophagy‐lysosome and ubiquitin‐proteasome pathways. Herein, we evaluated whether TUG1 overexpression could induce autophagy in HUVECs, and found that the autophagy marker LC3 was not significantly altered following TUG1 overexpression (Figure S5D, Supporting Information). Conversely, the proteasome inhibitor MG132 could partially reverse the TUG1 overexpression‐induced HuR protein downregulation in HUVECs (Figure 5E). This finding suggests that TUG1 overexpression in HUVECs caused HuR degradation via the proteasome pathway. Subsequently, we examined whether HuR was ubiquitinated following TUG1 upregulation and found that the ubiquitin‐modified HuR protein was significantly downregulated in HUVECs following TUG1 overexpression (Figure 5F). These findings collectively suggest that TUG1 promoted HuR degradation through the ubiquitin‐proteasome pathway.

### 2.7 TUG1 Could Reverse the Angiogenesis Role of HuR Both In Vivo and In Vitro

**Figure 6:**
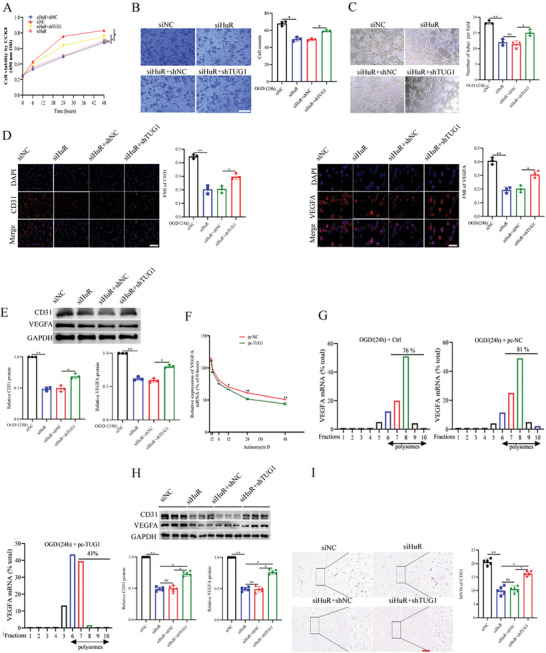
The angiogenesis role of HuR could be reversed by TUG1 in vivo and in vitro. A–C) The proliferation, migration, and tube formation ability were measured respectively (*n* = 3). Scar bar = 25 um. D) The levels of CD31 and VEGFA in HUVECs were detected by the immune‐fluorescence (*n* = 3). Scar bar = 25 um. E) The protein levels of CD31 and VEGFA in HUVECs were analyzed by the WB (*n* = 3). F) VEGFA mRNA stability was measured by RT‐qPCR after HUVECs were exposed to Actinomycin D (*n* = 3). G) Polysomes assays were performed in HUVECs (*n* = 3). VEGFA mRNA was measured with Taqman probes and presented here as the percentage in each fraction. H) The expression of CD31 and VEGFA were detected with WB (*n* = 5). I) The microvessel density was detected by the immunohistochemical analysis (*n* = 5). Scar bar = 25 um. Data are presented as means ± SD. ^*^ *p* < 0.05, ^**^ *p* < 0.01 as calculated by one‐way ANOVA with Bonferroni's multiple comparison post hoc test.

We assessed the correlation between TUG1 and HuR in vitro, revealing that TUG1 knockdown could increase the proliferation, migration, and tube formation abilities of OGD/R‐treated HUVECs with HuR knockdown (**Figure** **6**A–C). Furthermore, the IF analysis results revealed that CD31 and VEGFA levels were higher in both the HuR and TUG1 knockdown groups compared to the HuR knockdown alone group (Figure 6D). Additionally, HuR knockdown inhibited the CD31 and VEGFA protein levels, while TUG1 knockdown exerted a contrary effect (Figure 6E). These findings suggest that HuR promoted angiogenesis in OGD/R‐induced HUVECs, a phenomenon that was reversed by TUG1 knockdown.

To further explore the angiogenesis inhibition role of TUG1, the VEGFA mRNA levels were detected following actinomycin D treatment. According to the results, TUG1 overexpression dropped the half‐life of VEGFA mRNA (Figure 6F). Furthermore, the polysome profiling assay revealed a significant difference in VEGFA mRNA abundance in the polysomal fractions between the control and TUG1 overexpression groups (Figure 6G). These findings suggest that TUG1 inhibited VEGFA mRNA stability in OGD/R‐treated HUVECs.

Lastly, we detected the protein levels to confirm the role of TUG1 and HuR in angiogenesis in CIRI mice, revealing higher expression of CD31 and VEGFA following concurrent knockdown of HuR and TUG1, compared to HuR knockdown alone (Figure 6H). Furthermore, the IHC analysis results revealed a higher vessel density when both TUG1 and HuR were knocked down compared to when only HuR was knocked down (Figure 6I). These findings collectively suggest that TUG1 could reverse the angiogenesis role of HuR both in vivo and in vitro.

## 3 Discussion

Increasing research evidence has posited that promoting angiogenesis could enhance poststroke functional recovery.^[^ ^19^ ^]^ Herein, compared to normal cerebral tissues, CIRI tissues showed a higher TUG1 expression, which correlated with increased infarct volume. Furthermore, TUG1 knockdown improved neurological function in CIRI mice, a phenomenon that correlated closely with the increased vessel density in the ischemic infarct zone. Moreover, HuR promoted angiogenesis and alleviated CIRI, phenomena that correlated with VEGFA mRNA stability.

Some RBPs were previously implicated in the pathological mechanisms of cerebrovascular diseases.^[^ ^20^, ^21^ ^]^ For instance, a previous study found that HuR could be modulated by cystathionine γ‐lyase‐induced S‐sulfhydration, and correlated with endothelial dysfunction onset.^[^ ^22^ ^]^ Furthermore, HuR was reported to regulate the production of angiogenesis‐linked proteins under stimulatory conditions.^[^ ^23^ ^]^ Additionally, HuR could contribute to ischemia, matrix protein accumulation, and angiogenesis in the kidneys.^[^ ^24^ ^]^ Herein, HuR promoted angiogenesis and alleviated dysfunction in CIRI mice.

As a nucleoplasmic shuttle protein, HuR is typically localized in the nucleus, protecting target mRNA from degradation.^[^ ^25^ ^]^ Notably, the stimulus‐dependent translocation of HuR from the nucleus to the cytoplasm is considered a critical step in mRNA stability regulation at the post‐transcriptional level.^[^ ^26^ ^]^ Furthermore, multiple signaling pathways, such as Mitogen‐Activated Protein Kinases (MAPKs) have been established to target HuR.^[^ ^27^, ^28^ ^]^ It is also noteworthy that in response to genotoxic stress, tmicroRNA‐125b interacted with HuR, regulating p53 mRNA translation.^[^ ^29^ ^]^ Additionally, HuR‐mediated translational control of NOX4 was implicated in the fibrosis development of the glomerular microvascular bed.^[^ ^30^ ^]^ Moreover, improved VEGFA mRNA stability has been considered a vital angiogenesis factor.^[^ ^31^ ^]^ Herein, OGD/R treatment triggered HuR cytoplasmic accumulation and enhanced its binding with VEGFA mRNA, a phenomenon that correlates with its pro‐angiogenesis capabilities in CIRI.

Studies have also reported that RBPs could bridge coding RNAs and non‐coding RNAs, thus regulating the expression of targeted coding genes.^[^ ^32^ ^]^ Using a high‐throughput peripheral blood‐based approach, a previous study detected thousands of differentially expressed lncRNAs in patients with cerebral ischemia.^[^ ^33^ ^]^ Most of the lncRNAs were associated with specific ischemic processes, including angiogenesis, which is responsible for post‐ischemic repair and regeneration.^[^ ^34^ ^]^ Besides regulating target genes directly, lncRNAs also interact with RBPs to influence target mRNA stability, thus controlling gene expression.^[^ ^35^ ^]^ Herein, we confirmed that TUG1 suppressed angiogenesis and aggravated CIRI in vivo. Furthermore, TUG1 knockdown partially rescued the HuR knockdown‐induced inhibition of the angiogenesis capacity of HUVECs. Additionally, the RNA pull‐down and RIP assays revealed the TUG1‐HuR interplay, with the CLIP assay confirming direct TUG1‐HuR binding. Furthermore, TUG1 overexpression inhibited VEGFA mRNA stability.

According to research, lncRNAs localized in the nucleus might co‐regulate transcription or chromatin structure in cis or trans, while those in the cytoplasm could regulate translation, stabilize mRNA, and function as sponges for miRNAs.^[^ ^36^ ^]^ For instance, TUG1 knockdown could alleviate CIRI‐induced apoptosis by targeting the miR‐410/FOXO3 axis, regulating angiogenesis.^[^ ^32^, ^37^ ^]^ Although the miRNA sponge function of lncRNAs is somewhat controversial, lncRNA‐protein interaction patterns are more interesting and complex than those of lncRNA‐miRNA interactions.^[^ ^38^ ^]^ Herein, HuR mRNA expression, rather than HuR protein expression, decreased significantly following TUG1 overexpression. Furthermore, TUG1 overexpression in HUVECs induced HuR degradation via the ubiquitin‐proteasome pathway. Moreover, suppressing the nuclear translocation of HuR inhibited angiogenesis, a phenomenon that undoubtedly supplemented the novel mechanism of TUG1 in regulating angiogenesis.

Ubiquitination is a post‐translational modification mechanism that mediates protein degradation via the proteasome pathway.^[^ ^39^ ^]^ Herein, TUG1 regulated the ubiquitin degradation and nuclear translocation of HuR, highlighting the role of lncRNAs in post‐transcriptional gene expression regulation. Consistent with our results, a previous study reported that catalytic action‐induced compounds upregulated HuR by inhibiting the ubiquitin‐proteasome pathway, thus triggering autophagic activation and promoting autophagic ferritin degradation, which, in turn, led to ferroptosis.^[^ ^40^ ^]^ Another study reported that triciribine induced the translocation of HuR from the nucleus to the cytoplasm in an ERK‐dependent manner, thus stabilizing low‐density lipoprotein receptor mRNA in HepG2 cells,^[^ ^41^ ^]^ a phenomenon that also aligned with our findings. Moreover, our CLIP assays revealed that the specific binding sites of HuR and VEGFA mRNA were different from those between HuR and TUG1, indicating that there was noncompetitive binding among them. Additionally, TUG1 demonstrated angiogenesis effects in CIRI mice by suppressing the nuclear translocation of HuR and promoting its ubiquitination, phenomena that correlated with VEGFA mRNA stability.

Owing to advancements in the development of pro‐angiogenesis drugs, functional improvements have been realized in pertinent patient groups. Particularly, small‐molecule inhibitors against HuR have been employed in various cancers, including Non‐Small Cell Lung Cancer (NSCLC).^[^ ^42^, ^43^ ^]^ Furthermore, a modified HuR protein was found to promote retinal pathology in diabetic animals.^[^ ^44^ ^]^ Additionally, HuR could improve the stabilization of target mRNA levels, contributing to Diabetic Nephropathy (DNP) in kidneys.^[^ ^45^ ^]^ Herein, TUG1 knockdown and HuR overexpression improved angiogenesis, highlighting a potential avenue for developing targeted drugs that could be used for CIRI treatment. Moreover, several small‐molecule inhibitors that could disrupt the HuR‐mRNA complex have been reported. One such inhibitor, KH‐3, could disrupt HuR‐FOXQ1 mRNA interactions, hindering breast cancer cell growth and invasion.^[^ ^46^ ^]^ Furthermore, disrupting lncRNA MAARS‐HuR interactions could reduce macrophage apoptosis and vascular remodeling in advanced plaques across various chronic diseases.^[^ ^47^ ^]^ Therefore, TUG1‐HuR interactions could be leveraged to develop targeted treatments.

Despite its valuable insights, this study had some limitations. First, only short‐term effects of TUG1 and HuR on angiogenesis were examined in MCAO mice due to the model's relatively quick recovery time window, necessitating additional research to examine the long‐term effects. Second, we relied heavily on the fact that TUG1 has been reported as a miRNA sponge in cerebrovascular diseases and did not consider the possibility that other regulatory mechanisms could also impact TUG‐HuR interactions.^[^ ^48^ ^]^ Lastly, our study explored the roles of HuR and TUG1 in angiogenesis at specific time points following OGD or MCAO in the designed models, introducing a potential gap regarding clinical practices.

## 4 Conclusion

**Figure 7:**
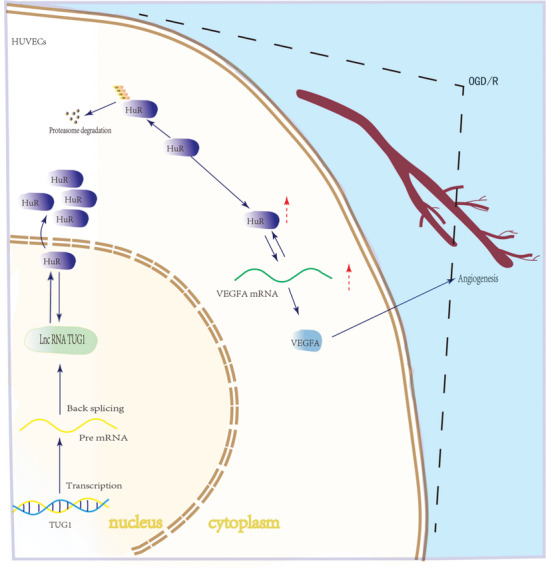
A schematic illustration of the molecular mechanism of TUG1 in regulating the angiogenesis of HUVECs. TUG1 targeted to HuR by promoting its ubiquitination and inhibiting its nuclear translocation, subsequently restricting the stability of VEGFA mRNA and inhibiting HUVECs angiogenesis.

Herein, we found that TUG1 inhibited angiogenesis in OGD/R‐induced HUVECs and exacerbated CIRI in mice. Mechanistically, TUG1 binds to HuR, inhibiting its nuclear translocation and protein levels, thus suppressing VEGFA mRNA. Specifically, TUG1 promoted HuR ubiquitination, thus suppressing its protein level (**Figure** **7**). Overall, TUG1 could be a novel therapeutic target for CIRI.

## 5 Experimental Section

### Oxygen Glucose Deprivation/Reoxygenation (OGD/R) Treatment

First, Human Umbilical Vein Endothelial cells (HUVECs; Cell Bank Type Culture Collection of the Chinese Academy of Sciences) were cultured in an Extracellular Matrix (ECM) medium supplemented with 10% Fetal Bovine Serum (FBS), and antibiotics (100 µg mL^−1^ streptomycin and 100 µg mL^−1^ penicillin), and then incubated at 37 °C in a 5% CO_2_ incubator. Normal HUVECs were then removed from the medium and incubated in Dulbecco's Modified Eagle Medium (DMEM) without glucose at 37 °C, 95% N_2_, and 5% CO_2_ across different times. The cells were then incubated in normoxic environments for 24 h to restore glucose and oxygen supply.

### Western Blot (WB) Analysis

First, total protein, which was extracted using RIPA lysis buffer, was separated through Sodium Dodecyl Sulfate‐Polyacrylamide Gel Electrophoresis (SDS‐PAGE) and then transferred onto Polyvinylidene Difluoride (PVDF) membranes (Millipore ISEQ00010). The membranes were first incubated with primary antibodies, and then with secondary antibodies. The primary antibodies included anti‐HuR (1:1000; Proteintech), anti‐VEGFA (1:1000, Proteintech), Lc3A/B (1:500; Abcam), and anti‐CD31 (1:500; Abcam). Ultimately, the protein bands were visualized using a stable western chemiluminescent Horseradish Peroxidase (HRP) substrate.

### Real‐Time Quantitative Polymerase Chain Reaction (RT‐qPCR)

First, RNA was extracted using TRIzol reagent (TaKaRa Biotech, Japan) before performing cDNA synthesis with the Prime ScriptTM RT reagent (TaKaRa Biotech). The relative gene expression levels were then determined using the SYBR Premix Ex Taq system (TliRNaseH Plus). The RT‐qPCR experiment was conducted using the StepOnePlus RT‐qPCR system (ABI; CA, USA). Table S1 (Supporting Information) details the primers used.

### Enzyme‐Linked Immunosorbent Assay (ELISA)

Following the manufacturer's instructions, the VEGFA ELISA Kit (Sino Biological, 11066‐R010) was used to detect in vitro VEGFA protein levels in the supernatants of HUVECs.

### Immunofluorescence (IF) Assay

The IF assay was conducted as outlined in previous research.^[^ ^49^ ^]^ Briefly, after fixing for 30 min in a 4% Paraformaldehyde (PFA) solution, the cells were permeabilized for 10 min with 0.1% Triton X‐100. Following that, the cells were blocked for an hour with 1% Bovine Serum Albumin (BSA) and then treated with the designated primary antibodies overnight. Subsequently, the cells were incubated with fluor‐labeled secondary antibodies. Finally, cell nuclei were DAPI‐stained and imaged using an Olympus BX51 or FV1200 confocal microscope.

### Nucleus–Cytoplasm Fractionation

Cytoplasmic and nuclear proteins from HUVECs were isolated using the Subcellular Protein Fractionation Kit for Cultured Cells (Thermo Fisher Scientific Inc., #78840) following the manufacturer's instructions.^[^ ^50^ ^]^

### Cell Transfection

The TUG1 overexpression vector and its empty vector, shTUG1 and its scrambled siRNA (shNC), the HuR overexpression vector and its empty vector, and siHuR and its scrambled siRNA (siNC) were sourced from GenePharma (Shanghai, China). After seeding the cells at appropriate densities into 6‐well plates, they were transfected with related plasmids using the Lipofectamine 2000 reagent (Invitrogen). The transfected cells were cultured for an additional 24 h and then subjected to OGD/R treatment.

### Tube Formation Assay

First, a 24‐well plate was precoated for 30 min with Matrigel (BD Biosciences, USA), seeded with HUVECs, and then incubated with DMEM and 15% FBS for 24 h at 37 °C. Tube formation was visualized using a microscope.

### Cell Counting Kit‐8 (CCK‐8) Assay

After transfection with relevant plasmids, the cells were grown for a specified period. Subsequently, 180 uL new culture media and 20 uL CCK‐8 solution (Beyotime, Shanghai, China) were added to each well and incubated for 2 h at 37 °C. Absorbance was then measured at 450 nm using a microplate reader (Bio‐Rad, CA, USA). Finally, proliferation curves were plotted.

### Transwell Assay

First, the bottom Transwell chambers were filled with DMEM containing 10% FBS (600 uL). After suspension in 100 uL serum‐free media, the HUVECs were seeded into the top chamber. After a 24 h incubation period at 37 °C, the membranes containing migrating cells were fixed and stained with crystal violet. The migrated HUVECs were then counted under an inverted light microscope. The number of migrating cells was calculated by counting the number of cells from ten randomly selected fields.

### The RNA Stability Assay

Herein, mRNA stability was assessed using the Actinomycin D (ActD) assay as outlined in previous research.^[^ ^51^ ^]^ After transfection with related plasmids, the cell medium was supplemented with ActD (a *de novo* transcription inhibitor) at a final concentration of 5 ug ml^−1^. Following that, VEGFA mRNA was assessed using the RT‐qPCR assay.

### Polysome Assay

The polysome test was performed as outlined in previous research.^[^ ^52^ ^]^ First, post‐nuclear supernatants were separated into ten fractions with a 15%–40% sucrose gradient via centrifugation at 200 000 × g. Quantitative RT‐qPCR was then performed with total RNA that was extracted using the TRIzol technique.

### RNA Immunoprecipitation (RIP) Assay

The cells were first collected and suspended in lysis buffer (Beyotime Biotechnology, China). Antibodies against HuR and IgG (Beyotime Biotechnology, Cat#A7031) were then added to the whole lysate. Protein A/G plus‐agarose (Santa Cruz Biotechnology, Cat#sc‐2003) was employed to extract RNA‐protein complexes and was rinsed four times with lysis buffer. Finally, the precipitated RNA was quantified using RT‐qPCR.

### RNA Pull‐Down Assay

The RNA pull‐down experiment was performed using the RNA pull‐down kit (BersinBio, Guangzhou, China). First, the cell lysates were treated with a biotinylated RNA probe for TUG1 (Beijing Tsingke Biotech Co., Ltd). Subsequently, the solution was mixed with streptavidin‐modified magnetic beads (Invitrogen, 11205D) and incubated for 48 h. Proteins were then isolated and evaluated through WB analysis.

### Fluorescence In Situ Hybridization (FISH) Assay

First, the VEGFA sequence (5′‐ AGTCTCCTCTTCCTTCATTTCAGGTTT‐3′) was identified using the Cy3‐labeled VEGFA mRNA probe (Beijing Tsingke Biotech Co., Ltd), while the 6‐FAM‐labeled TUG1 mRNA probe (Beijing Tsingke Biotech Co., Ltd) was used to identify the TUG1 sequence (5′‐ GCTATTTGGCTTGAGGACTCTGACTTC‐3′). The FISH assay was performed as outlined in previous research.^[^ ^53^ ^]^ Briefly, the cells were first treated with a complex digestive solution containing proteinase K and pepsin, denatured at 75 °C for 5 min, wet and dark hybridized overnight at 42 °C, and treated with a probe hybridization solution. After washing with 0.4 SSC and PBS, the nuclei were DAPI‐stained and images were captured using a Confocal Laser Microscope (CLM).

### Cross‐Linking Immunoprecipitation (CLIP)

The CLIP assay was performed as previously reported.^[^ ^54^ ^]^ Briefly, the UV crosslinker (Thermo Fisher Scientific, US) (wavelength 260 nm) was first employed to irradiate 2 * 10^7^ HUVECs. The cells were then lysed using a RIPA buffer and co‐incubated with protein A/G magnetic beads for ≥ 4 h at 4 °C. Subsequently, the immune complexes were eluted with a 50 mm Tris‐HCl solution for 20 min at 60 °C. The RNA was extracted using chloroform to separate it from the immunocomplexes. Finally, the expression levels of the target genes were determined and evaluated using RT‐qPCR.

### Co‐Immunoprecipitation (IP) Assay

First, the HUVECs were collected and incubated with Protein‐A/G MagBeads (Yeason, Shanghai, China) and appropriate antibodies. After incubating overnight, the immune complexes were washed and centrifuged. The proteins were then detected via WB analysis with an anti‐ubiquitin antibody, following the manufacturer's instructions. Anti‐HuR antibodies were used in the Co‐IP tests.

### Cycloheximide (CHX) and Proteasome Inhibitor (MG132) Assays

Notably, CHX functions as a eukaryotic protein synthesis inhibitor and could be employed to evaluate protein stability. After transfection with pc‐TUG1 plasmids using Lipofectamine 2000 (from Invitrogen) for 24 h, the HUVEC cells were treated with CHX (20 µg/ml) (Genview, Beijing, China) for 0, 6, and 12 h. We observed that MG132 could efficiently inhibit the activity of the 26S proteasome complex targeted at ubiquitin‐binding proteins. Consequently, HUVECs were transfected with pc‐TUG1 plasmids for 30 h, followed by a 12 h treatment with MG132 (10 µM) (MedChemExpress, New Jersey, USA). The proteins were then detected.

### Middle Cerebral Artery Occlusion (MCAO) Model

Seven‐week‐old C57BL/6J male mice were deeply anesthetized with isoflurane and used to establish the MCAO models.^[^ ^55^ ^]^ Briefly, the mice were administered with 2% isoflurane in oxygen, followed by 1.0–1.5% isoflurane in 70% N2O and 30% O2. The anesthesia was maintained using a small‐animal anesthetic device. The common carotid artery and the left external carotid artery (ECA) were separated via a midline neck incision. An incision was made in the arterial wall, and a silicone rubber monofilament was inserted into the ECA following the temporary occlusion of the common carotid artery (CCA) and internal carotid artery (ICA). A monofilament was gradually advanced into the MCA via the left ICA. After 1h of occlusion, the monofilament was removed, and the ECA was permanently ligated. In the sham group, the MCA was blocked in mice through the insertion of the monofilament only. Subsequently, the monofilament was withdrawn and blood flow was quickly restored.

### Intracerebroventricular Administration

Related plasmids (GenePharma, Shanghai) were diluted with transfection reagent (entranser, Engreen Biosystem) and were injected before MCAO by intracerebroventricular injection as previously described.^[^ ^56^ ^]^ Briefly, mice were deeply anesthetized, and the specified plasmids were microinjected into the left lateral ventricle at a total volume of 3.0 µL, delivered at a controlled rate of 0.5 µL min^−1^ using a pump. After intracerebroventricular injection, the microsyringe was left in place for 10 min after which it was slowly withdrawn. For mice in the sham group, a burr hole was created followed by injection of an equivalent volume of saline. Throughout the procedure, the mice were maintained under anesthesia.

### Triphenyltetrazolium Chloride (TTC) Staining

Brain tissues were obtained and split into coronal sections, incubated for 30 min at 37 °C in a 2% TTC solution, and transferred into a 4% paraformaldehyde solution and fixed. The extent of infarction in each section was quantified using Image J software, as previously described in our methods.^[^ ^57^ ^]^

### Brain Water Content Analysis

The wet weights of the right and left hemispheres were measured as previously described.^[^ ^58^ ^]^ To record the dry weights, the brains were dried at 75 °C for 48 h and measured. The percentage of water content in cerebral tissues was calculated using the following formula: [(wet weight—dry weight)/wet weight] 100%.

### Modified Neurological Severity Score (mNSS)

Neurological impairments were evaluated using the mNSS after MCAO following a previous guideline.^[^ ^55^ ^]^ The motor, sensory, refex, and balance domains were determined based on the mNSS test. Higher results on this exam, which ranged from 0 to 12, indicated more severe neurological abnormalities.

### Immunohistochemistry (IHC) Assay

The brain regions surrounding the MCA region were collected and embedded in paraffin after fixation in 4% paraformaldehyde. The 5 µm‐thick tissue slices were incubated with primary antibodies overnight at a temperature of 4 °C. After staining, the sections were rinsed with PBS and then incubated with a secondary antibody at room temperature for 1 h. The staining was completed using diaminobenzidine as the chromogen. Images were captured and semi‐quantitative evaluation was performed across a minimum of five random fields using Image‐Pro Plus 6.0 software.

### Statistical Analysis

Statistical analyses were performed using GraphPad Prism 8.0 software. All data were presented as mean ± Standard Deviation (SD). Multiple comparisons were performed using one‐way ANOVA, followed by Bonferroni's post hoc multiple comparison test. Non‐normally distributed variables were evaluated using Mann‐Whitney U rank‐sum tests, while normally distributed variables were evaluated using the student's t‐test. Results with *p* < 0.05 were considered statistically significant.

### Ethics Approval and Consent to Participate

This study was approved by the Laboratory Animal Ethics Committee of Wuhan University People's Hospital (ID number: WDRM20220301C).

## Conflict of Interest

The authors declare no conflict of interest.

## Author Contributions

H.J and Q.C. contributed equally to this work as co‐first authors. All of them have contributed significantly and are in agreement with the content of the manuscript. CQX and LF have contributed to study conception and design. JHX has contributed to result interpretation and manuscript drafting. CQ and HPD have contributed to the statistical analysis. LF has contributed to interpretation of the study results and critical revision for important intellectual contents. All authors read and approved the final manuscript.

## Supporting information



Supporting Information

## Data Availability

The data that support the findings of this study are available from the corresponding author upon reasonable request.
