# Supplementary material for: LncRNA TUG1 Repressed Angiogenesis by Promoting the Ubiquitination of HuR and Inhibiting Its Nuclear Translocation in Cerebral Ischemic Reperfusion Injury
Source: Adv Sci (Weinh). 2025 Jan 31;12(12):2413333. doi: 10.1002/advs.202413333 (PMC11948051; doi:10.1002/advs.202413333)
Supplement: Supplementary file 1 — Supporting Information [file ADVS-12-2413333-s001.docx]

**Supplementary Figure 1**

**
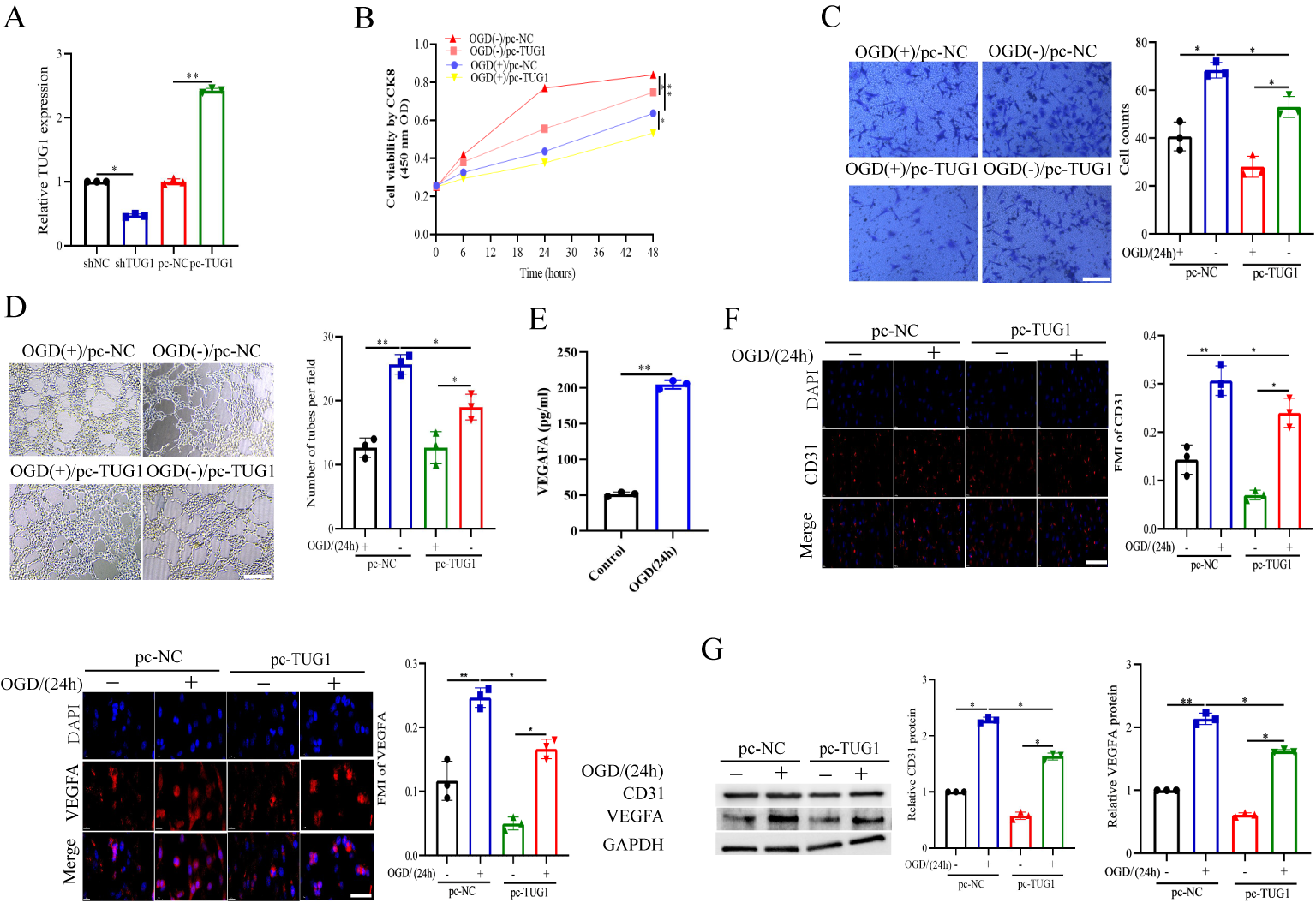
**

**Figure S1** **Up-regulated TUG1 inhibited angiogenesis *in vitro*.** (A) The knockdown and up-regulation of TUG1 was confirmed with RT-qPCR (n = 3). (B-D) The proliferation, migration and tube formation ability were measured respectively (n = 3). Scar bar = 25um. (E) Measurement of VEGFA secreted by HUVECs (n = 3). (F) The levels of CD31 and VEGFA in HUVECs were detected by the immune-fluorescence (n = 3). Scar bar = 25um. (G) The levels of CD31 and VEGFA in HUVECs were analyzed with WB. Data are presented as means ± SD. **p* < 0.05, ***p* < 0.01 as calculated by one-way ANOVA with Bonferroni’s multiple comparison post hoc test.

**Supplementary Figure 2**

**
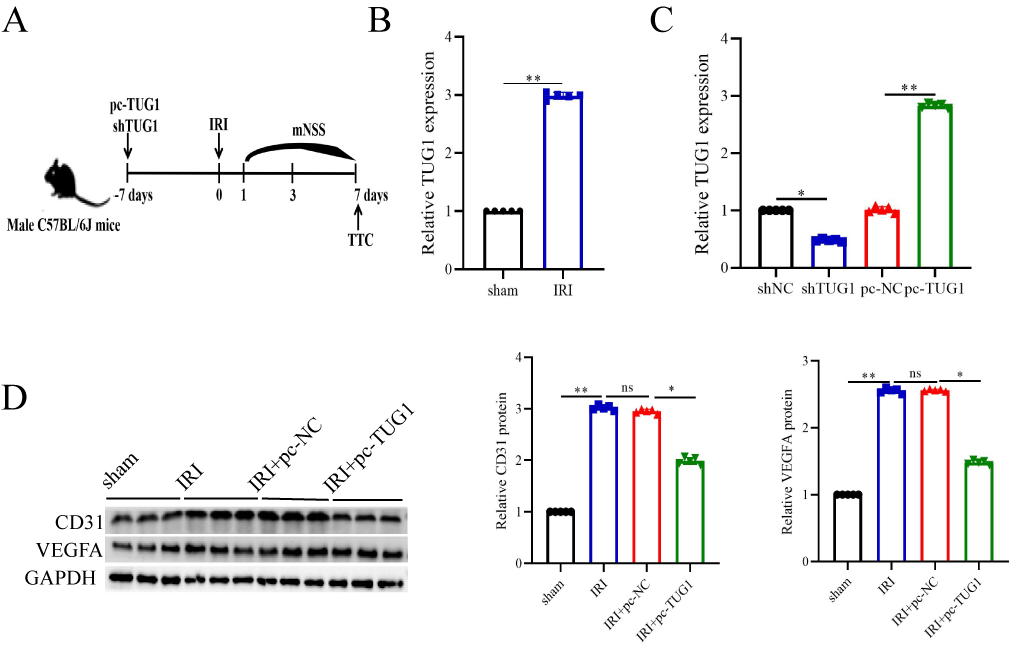
**

**Figure S2 Up-regulated TUG1 inhibited angiogenesis *in vivo.*** (A) The time schedule for the related treatment on the premise of the knockdown or up-regulation of TUG1. (B) The up-regulated HuR levels in mice IRI group comparing with the sham group (n = 5). (C) The knockdown and overexpression of TUG1 were confirmed with RT-qPCR (n = 5). (D) The protein of CD31 and VEGFA were analyzed by WB (n = 5). Data are presented as means ± SD. **p* < 0.05, ***p* < 0.01 as calculated by Mann–Whitney test (for B) and one-way ANOVA with Bonferroni’s multiple comparison post hoc test (for C, D).

**Supplementary Figure 3**

**
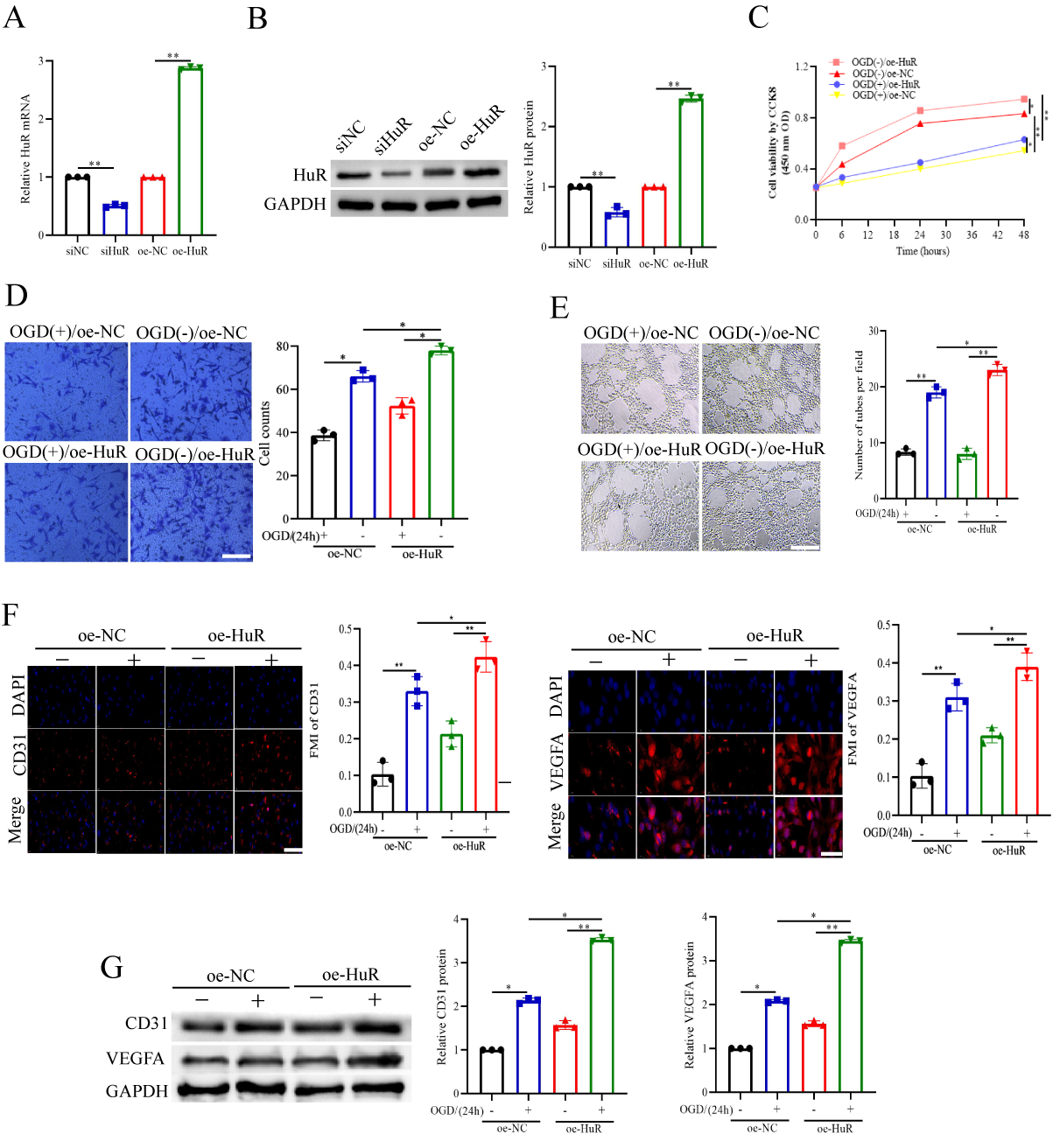
**

**Figure S3 Up-regulated HuR promoted angiogenesis *in vitro.*** (A, B) The knockdown and up-regulation of HuR was confirmed with RT-qPCR and WB (n = 3). (C-E) The proliferation, migration and tube formation ability were measured respectively (n = 3). Scar bar = 25um. (F) The levels of CD31 and VEGFA in HUVECs were detected by the immune-fluorescence (n = 3). Scar bar = 25um. (G) The levels of CD31 and VEGFA in HUVECs were analyzed by WB (n = 3). Data are presented as means ± SD. **p* < 0.05, ***p*< 0.01 as calculated by one-way ANOVA with Bonferroni’s multiple comparison post hoc test.

**Supplementary Figure 4**


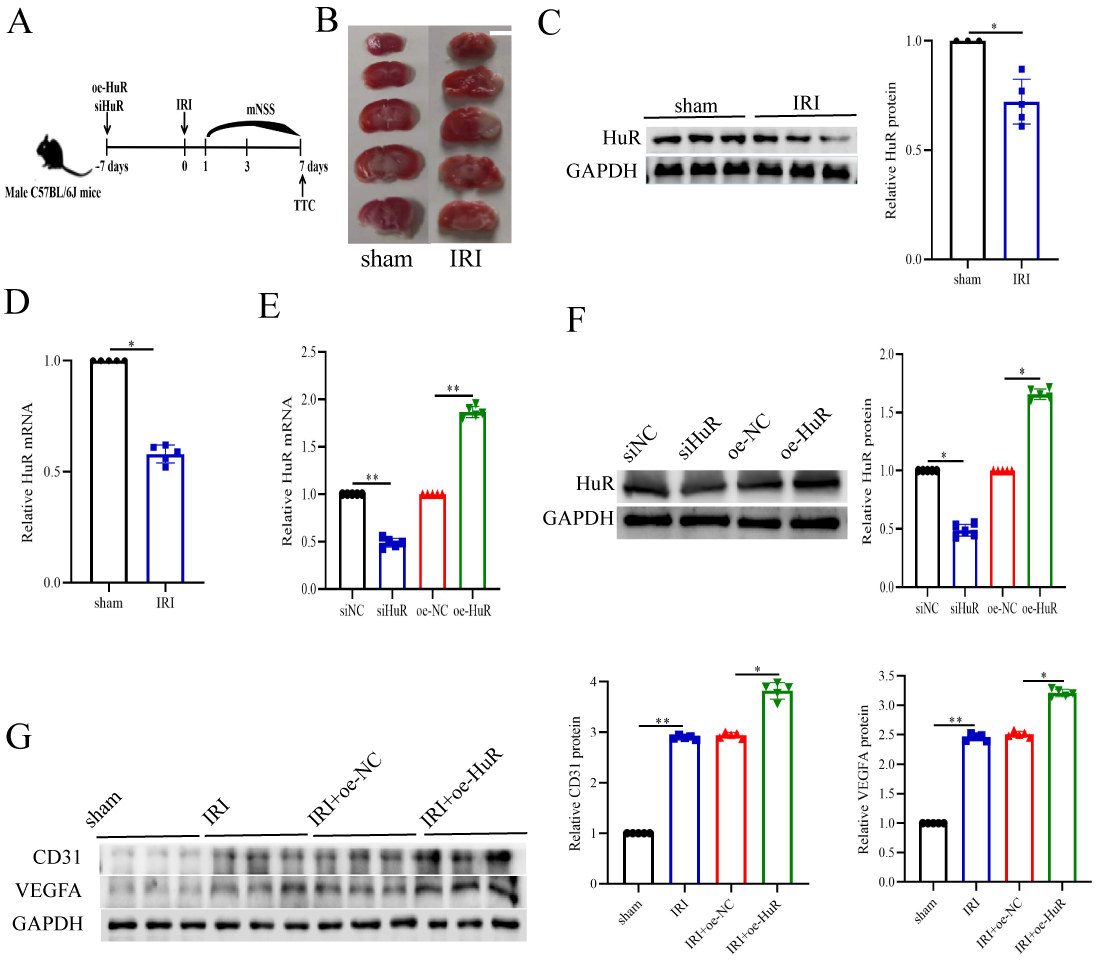


**Figure S4 Up-regulated HuR promoted angiogenesis *in vivo.*** (A) The time schedule for the related treatment on the premise of the knockdown or up-regulation of TUG1. (B) TTC-stained brain slices in sham and IRI group (n = 5). Scale bars =5 mm. (C, D) The down-regulated HuR levels in mice IRI group comparing with the sham group (n = 5). (E,F) The knockdown and overexpression of HuR were confirmed with RT-qPCR and WB (n = 5). (G) The levels of CD31 and VEGFA were analyzed by WB (n = 5). Data are presented as means ± SD. **p* < 0.05, ***p*< 0.01 as calculated by Mann–Whitney test (for C and D) and one-way ANOVA with Bonferroni’s multiple comparison post hoc test (for E- G).

**Supplementary Figure 5**

**
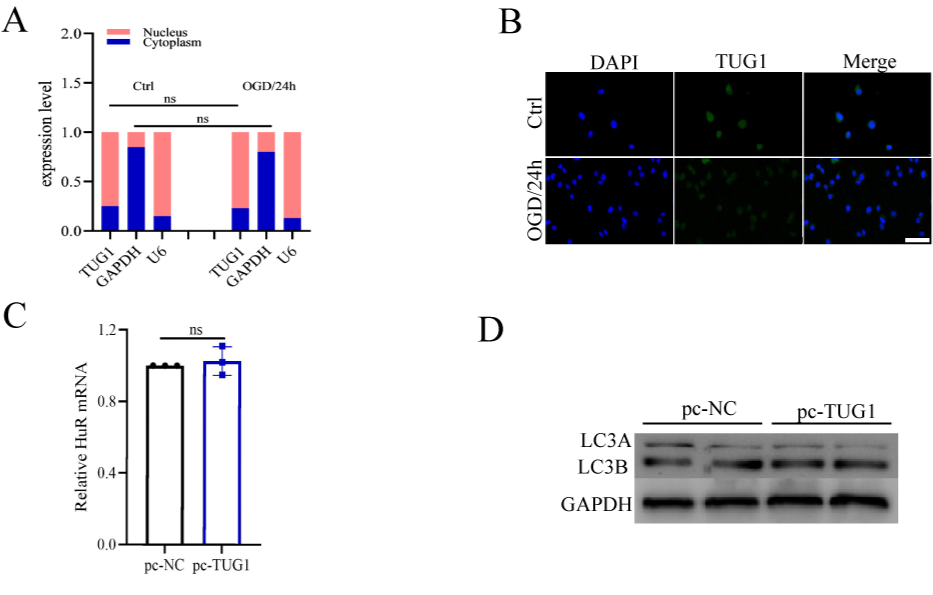
**

**Figure S5 TUG1 inhibited the nuclear translocation and promoted the ubiquitination degradation of HuR.** (A, B) The localization of TUG1 in HUVECs was determined by subcellular fractionation assay and RNA FISH (n = 3). Scale bar=50µm. (C) The mRNA levels of HuR in HUVECs after the up-regulation of TUG1 (n = 3). (D) Expression of LC3 in HUVECs after the up-regulation of TUG1 (n = 3). Data are presented as means ± SD. ns as calculated by one-way ANOVA with Bonferroni’s multiple comparison post hoc test.

**Supplementary Figure 6**


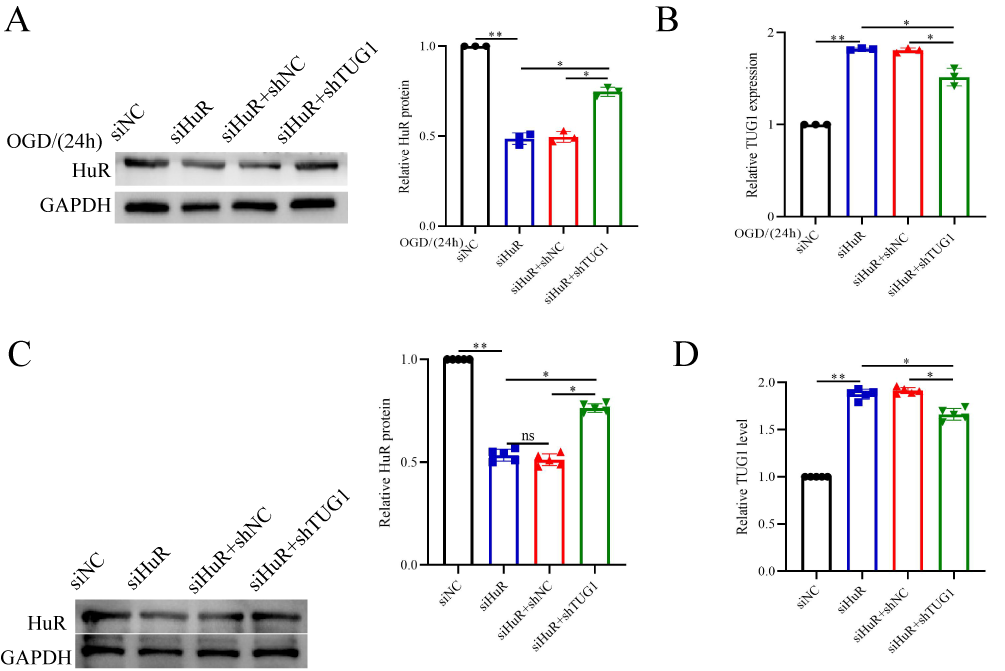


**Figure S6 The knockdown of TUG1 and HuR were confirmed in vivo and *in vitro*.** (A, B) The knockdown of HuR and TUG1 were confirmed in vitro (n = 3). (C, D) The knockdown of HuR and TUG1 were confirmed in vivo (n = 5). Data are presented as means ± SD. **p* < 0.05, ***p* < 0.01 as calculated by one-way ANOVA with Bonferroni’s multiple comparison post hoc test.

**Supplementary Table S1** PCR primers sequences

| RNA | Forward sequence | Reverse sequence |
| --- | --- | --- |
| HuR | 5'-ACCATGACCCAGAAGGACGTAGA-3' | 5'-AAACTGGTAATTGCCTCTTCTGC-3' |
| CD31 | 5'-ATTGCAGTGGTTATCATCGGAGTG-3' | 5'-CTGGTTGTTGGAGTTCAGAAGTGG-3' |
| VEGFA | 5'-AGGGCAGAATCATCACGAAGT-3' | 5'-AGGGTCTCGATTGGATGGCA-3' |
| TUG1 | 5'-AATGGCACCCAGTGTAAAGCA-3' | 5'-AGCCTATTCACCACCAACCACA-3' |
| GAPDH | 5'-GGAAGCTTGTCATCAATGGAAATC -3' | 5'-TGATGACCCTTTTGGCTCCC-3' |
